# Oral Immunization of Chickens With Recombinant *Lactobacillus plantarum* Vaccine Against Early ALV-J Infection

**DOI:** 10.3389/fimmu.2019.02299

**Published:** 2019-10-02

**Authors:** Shenghua Wang, Na Geng, Dong Zhou, Yi Qu, Mengke Shi, Yuliang Xu, Kangping Liu, Yongxia Liu, Jianzhu Liu

**Affiliations:** ^1^College of Veterinary Medicine, Shandong Agricultural University, Tai'an, China; ^2^Shandong Provincial Engineering Technology Research Center of Animal Disease Control and Prevention, Shandong Agricultural University, Tai'an, China; ^3^Research Center for Animal Disease Control Engineering, Shandong Agricultural University, Tai'an, China; ^4^College of Veterinary Medicine, Northwest Agriculture and Forestry University, Yangling, China; ^5^Nanjing Entry-Exit Inspection and Quarantine Bureau, Nanjing, China

**Keywords:** ALV-J, gp85 protein, *Lactobacillus plantarum*, *pgsA*, oral immunization

## Abstract

In this study, a novel oral vaccine of recombinant *Lactobacillus plantarum* (*L. plantarum*) containing the gp85 protein was explored, and the effects of this vaccine on the prevention of subgroup J Avian Leukosis Virus (ALV-J) infection were assessed. In the current study, the gp85 protein of ALV-J was expressed on the surface of *L. plantarum* with the surface-display motif, *pgsA*, by constructing a shuttle vector *pMG36e:pgsA:gp85*. Surface localization of the fusion protein was verified by western blotting and flow cytometry. Subsequently, Specific Pathogen Free Hy-Line Brown layer chickens were orally vaccinated with the recombinant *L. plantarum* and presented with high levels of serum immunoglobulin G (IgG) and secretory immunoglobulin A (sIgA) titers in bile and duodenal-mucosal fluid. After challenged with ALV-J of a 3 × 10^3^ 50% tissue culture infective dose (TCID50), serum samples of the chickens were collected and viremia was analyzed. Results showed that, compared to the *L. plantarum* and PBS control group, the recombinant *L. plantarum* group showed a significant rise in antibody levels after inoculation, and provide improved protection against ALV-J according to viremia detection. These results indicate that oral immunization with the recombinant *L. plantarum* provided an effective means for eliciting protective immune response against early ALV-J infection.

## Introduction

The J Subgroup Avian Leukosis Virus (ALV-J) is a subgroup of retrovirus that was first reported and isolated from broiler chickens in the United Kingdom in the early 1990s (1). However, numerous cases of ALV-J infection and tumors in commercial layer chickens and broiler breeders have emerged in recent years (2–5). This resulted in serious economic losses to breeding farms, including growth retardation, high mortality, tumor production and cost for eradication. Therefore, here is an urgent need to take measures to protect chickens against ALV-J infection.

As we know, ALV-J spreads via vertical and horizontal infections, with horizontal transmission accounting for most ALV-J infections during the brood period (6). Vertical transmission from broiler breeders to progeny is frequent with ALV-J (7); this is due to the current lack of an effective vaccine against ALV. Consequently, researchers have been trying to explore a vaccine that will increase the proportion of positive antibodies against ALV through incubating breeder flocks, thereby reducing the chance of infection from infected chickens and improving the resistance of chickens by increasing ALV maternal antibodies to later infection. However, the inactivated vaccine has no significant effect, because the antigenicity of the inactivated virus is commonly destroyed (8). Furthermore, considering the complexity of ALV, the existing vaccine did not work well (9, 10). The glycoprotein (*gp*) with *Mr* of 85,000 (gp85) is encoded by the envelope (*env*) gene of ALV, and that the gp85 protein forms globular structures on the surface of the virus, and is closely associated with viral neutralization which determines the antigenicity of ALV-J (11). Previous researches have shown that the protein could be used as a vaccine to help ALV-J infect susceptible cells and induce the production of specific antibodies among infected chickens (12, 13). Therefore, this study set out to search for a live vehicle that could carry the surface protein gene *gp85* to prevent the ALV-J infection.

The mucosal immune barrier is the first line of defense of the immune system, and mucosal inoculation is a practical, non-invasive and efficacious method for the simultaneous induction of systemic and mucosal immune responses (14, 15). Oral vaccination is more favored and convenient than other routes of mucosal vaccination (16–18). The edible lactic acid bacteria, *lactobacillus plantarum*, is well-known as a safe candidate live vector vaccine to deliver antigen (17). Many recent investigations have analyzed and validated the potentiality of recombinant lactic acid bacteria for the delivery of heterologous antigens to the mucosal immune system (15, 16, 18–20).

Poly-γ-glutamate synthase A (pgsA) is a constituent protein of *Bacillus subtilis* polyglutamate synthetase (PGA), which can be used as a bacterial surface display element to immobilize the enzyme system on the surface of the cell membrane. In view of the characteristics of pgsA protein, it has been applied to various prokaryotic proteins on the surface display, in particular the lactic acid bacteria and other Gram-positive receptor strains (21). These findings provided a theoretical basis for studying the process of immobilizing exogenous proteins on the cell wall of *L. plantarum*.

In the current study, the recombinant *L. plantarum* harboring *pMG36e-pgsA-gp85* was constructed using genetic engineering technology, and then expressed on the surface of *L. plantarum*. The live recombinant *L. plantarum* was then used to orally vaccinate chickens. IgG and IgA antibodies against ALV-J, as well as viremia were detected to assess the effect of the recombinant *L. plantarum*.

## Materials and Methods

### Virus, Plasmids, and Antibodies

The *ALV-J-NX0101* strain, *pMD18T-env* recombinant vector (containing the *gp85* gene), gp85-specific mouse monoclonal anti-body (MAb JE9), *ALV-J* antibody test kit, and ALV P27 antigen enzyme-linked immunosorbent assay (ELISA) test kits (IDEXX USA Inc., Beijing, China) were donated by Prof. Zhizhong Cui. The *ALV* IgA antibody test kit was purchased from Lanpai Biotechnology Company (Shanghai, China). The *pgsA* gene and purified gp85 protein were stored in our laboratory. The *pMG36e* expression vector was obtained commercially (Invitrogen, Shanghai, China).

### Bacterial Strains and Growth Conditions

*L. plantarum* HQ542228 was purchased from China Center of Industrial Culture Collection (CICC) and grown anaerobically at 37°C, without agitation in MRS broth medium (250 g, Hopebiol, Qingdao, China). The *E. coli* strains were cultured at 37°C with continuous shaking in Luria–Bertani (LB) medium (250 g, Hopebiol).

### Animals

Hy-Line Brown layer chickens (1-day-old) were purchased from the Hylan Breeder Company (Shandong Province, China) and housed in a specific-pathogen-free environment at the Laboratory Animal and Resources Facility, Shandong Agricultural University. The animals had free access to water and commercial standard pellet diet from Liuhe Jingwei Farming and Animal Husbandry Co., Ltd. (Taian, China). Each chicken was confirmed negative for the ALV-J antibody and virus by ALV-J-antibody ELISA initiation of the experiment. The experimental procedure was approved by the Animal Care and Use Committee of the Shandong Agricultural University and performed in accordance with animal welfare and ethics guidelines (SDAUA-2016-037).

### Construction of Recombinant Strains

The primers were designed according to the *Bacillus subtilis pgsA* genes, complete cds sequence published in GeneBank (Number AB016245.1) by primer 5.0 and synthesized in Sangon Biotech (Shanghai, Co. Ltd). The forward and the reverse primer of the *pgsA* gene was 5′-CGAGCTCGCGAACTGAGCTTTCATGAAAAG-3′ and 5′-CTAGTCTAGACTATGATCAATATCAAACGTCA-3′, containing the *SacI* and *XbaI* sites (underlined) respectively, with the plasmid *T7-pgsBCA* as the template. PCR amplification was performed as follows: 95°C for 5 min, 30 cycles of 94°C for 30 s, 58°C for 30 s and 72°C for 72 s, and 72°C for 5 min of final extension. The PCR product was confirmed by DNA sequencing. The *gp85* gene was amplified using the forward primer (5′-TCATCTAGAGGGAGTTCATCTGTTG-3′) and the reverse primer (5′-TCCAAGCTTATTAGCGCCTGCTAC-3′) containing the *XbaI* and *Hind III* sites (underlined), respectively, with the *pMD18T-env* as the template. PCR amplification was performed as follows: 95°C for 5 min, 30 cycles of 94°C for 30 s, 56°C for 30 s and 72°C for 1 min, and 72°C for 5 min of final extension. The PCR product was confirmed by DNA sequencing.

Electroporation was performed according to the method of Josson et al. (22). Briefly, electrotransformation was performed in a 0.2 cm cuvette which was subjected to one single electric pulse (2.0 kV/cm, 200 Ω, 25 μF) using a Gene Pulser (Bio-Rad, Richmond, CA, USA). Recombinant strains were selected on MRS medium plates with 5 μg/ml of erythromycin (Ery; Sigma, St. Louis, MO, USA), which was incubated anaerobically at 37°C for 36 h. The recombinant *L. plantarum* containing the plasmid of the *pMG36e-pgsA-gp85* was identified via antibiotic selection and PCR experiments using the primers *pgsA* and *gp85*.

### Protein Expression and Identification (Immunoblotting and Flow Cytometry)

Sodium dodecyl sulfate polyacrylamide gel electrophoresis (SDS-PAGE) and western blotting were used to analyze the expression of the target protein, and western blotting was developed using the gp85-specific mouse monoclonal antibody JE9 as the primary antibody. Horseradish peroxidase (HRP)-conjugated goat anti-mouse IgG (Sigma, 1:5,000) was used as the secondary antibody.

For flow cytometry, *L. plantarum* cells were cultured in MRS broth overnight at 37°C. The cell pellets were sequentially incubated with gp85-specific mouse monoclonal antibody (1:800) and fluorescein isothiocyanate (FITC)-conjugated anti-mouse IgG secondary antibodies (1:5,000; Sigma). Finally, 3 × 10^4^ cells were analyzed with a FACS Calibur platform (Becton Dickinson, Oxnard, CA, USA) equipped with CellQuest software.

### Immunization of Chickens and Sample Collection

The layer chickens (120 in total) were randomly divided into three groups: control group, recombinant *L. plantarum* group and natural *L. plantarum* group. Each group contained 40 chickens that were kept in separated isolators receiving filtered positive-pressure air. The recombinant *L. plantarum* group was immunized intragastrically with recombinant strains of *L. plantarum* (harboring plasmid *pMG36e-pgsA-gp85*), while the chickens in the control and negative control group were inoculated with PBS and natural *L. plantarum*, respectively. The suspensions of strains for administration were prepared as follows: recombinant strains cultured overnight were collected by centrifugation at 3,000 × g for 10 min, washed three times with sterile PBS, and then resuspended in sterile PBS to a concentration of 5 × 10^9^ CFU/mL (400 μL/chicken). The control group and negative control group received equal doses of sterile PBS and natural *L. plantarum*. The immune protocol was administered on five consecutive days (days 1–5). A booster immunization was given between days 15 to 19, the second booster was given between days 29 to 33 and the third booster was given between days 43–47.

Blood samples were collected from the wing vein on Days 1 (before immunization), 7, 14, 28, 35, 42, and 49. Blood samples without additives were centrifuged and the serum was stored at −80°C for subsequent analysis.

To obtain bile and intestinal lavages samples, 3 chickens were randomly selected from each group, and were killed on Day 1 (pre-immune), 7, 14, 28, 35, 42, and 49. Moreover, using a previously described method based on that of Wu and Russell (23), intestinal lavage fluids were obtained by flushing the excised small intestine with 3 mL of PBS containing 50 mM ethylenediaminetetraacetic acid (EDTA) and 0.1 mg /mL of soybean trypsin-chymotrypsin inhibitor (Sigma). The contents were collected and retained on ice for processing, whereupon the fluids were vortexed and centrifuged at 650 × g for 10 min at 4°C. A 30 μL volume of 100 mM phenylmethylsulfonyl fluoride (PMSF, Sigma) was added to the supernatants before they were vortexed and spun at 27,000 × g for 20 min at 4°C. A further 20 μL of PMSF, 100 μL of fetal bovine serum (FBS), and 20 μL of 1% sodium azide (Sigma) were added to the supernatants before they were dispensed into aliquots and then stored at −80°C.

### ELISA

The anti-ALV-J antibody in serum, bile and duodenal lavages were analyzed by ELISA. Briefly, the titers of IgG were tested using an ALV-IgG ELISA Kit (IDEXX USA Inc., Beijing, China) according to the requirements of the specification. The optical density (OD) of the IgG were measured at 450 nm and determined by the sample-to-positive (S/P) method following the formula below: S/P = [(mean of OD_450nm_ of sample–mean of OD_450nm_ of negative control)]/[(mean of OD_450nm_ of positive control–mean of OD_450nm_ of negative control)]. The samples from each group were tested in three parallel trials, and the S/P values of any sample higher than 0.6 were considered as ALV-J antibody positive.

Furthermore, the commercial ALV-IgA antibody test kit (Lanpai Bio., Shanghai, China) was used to detect the positive ratio of the anti-ALV-J IgA in serum, bile and duodenal lavages according to the manufacture's protocol. The OD of the IgA was measured at 450 nm and determined by the Critical value (CUT OFF) method following the formula below: CUT OFF = (mean of OD_450nm_ of sample) + 0.15. The samples from each group were tested in three parallel trials, and the mean of OD_450nm_ of each sample higher than CUT OFF were considered to be ALV-J antibody positive. In our study, the test was considered effective if the mean of the negative control wells was <0.10 and the mean of the positive control was >1.00.

Indirect ELISA assay was used to assess the titers of anti-gp85 protein antibody. A 96-well ELISA plate was coated overnight with gp85 protein of appropriate concentration (4 μg/mL) and kept at 4°C. After blocking of the plates, the samples of serum, bile and duodenal lavages were used as the primary antibody and incubated at 37°C for 1 h. Then HRP-conjugated goat anti-chicken sIgA at a dilution of 1:2,000 (Sigma) at 37°C for 1 h was used to detect bound antibodies. Following washing the plate thice with PBS-0.05% Tween 20, tetramethylbenzidine (Qiagen, Germany) was added as the colorimetric substrate. Finally, the optical density (OD) was measured at 450 nm.

### Evaluation of Recombinant *L. plantarum* Protection Against ALV-J Challenge in Chickens

ALV-J NX0101 strain was selected as the challenge virus and transfected into chicken embryo fibroblasts (CEF) for amplification and detection of virulence. The TCID50 of ALV-J was judged following the method described by Fadly (24). All of the chickens were challenged intraperitoneally with 3 × 10^3^ TCID50 of the ALV-J NX0101 strain at Day 50. The plasma samples from the chickens were aseptically collected and then inoculated into the CEF cells in 24-wells plates. The cell supernatant of viremia was collected weekly to check for the presence of the virus using ALV P27 antigen ELISA test kits (IDEXX USA Inc.). The relative antigen titer level was determined by calculating the S/P ratio using the formula mentioned above. The samples from each group were tested in triplicate, and plasma samples with S/P ratios higher than 0.2 were considered virus-positive.

### Statistical Analysis

Statistical analysis was performed using the SPSS software (Version 11.0, SPSS Inc., USA). Meanwhile, one-way ANOVA was used to identify the significant values. All values were expressed as mean ± standard error of the mean (SEM). All measurements were replicated three times. The differences were considered significant when the *P*-value was <0.05.

## Results

### Construction of Recombinant Plasmids

**Figure 1:**
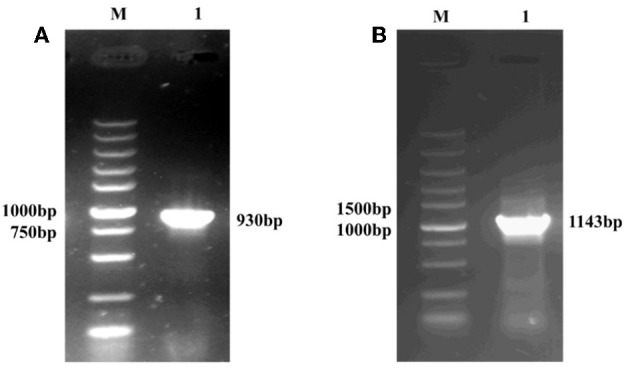
PCR amplification of the recombinant plasmid containing the gp85 gene **(A)** and the pgsA gene **(B)**, M: DNA Maker; **(A)** 1: the PCR product predicted as 930 bp. **(B)** 1: the PCR product predicted as 1,143 bp.

The amplification of the *gp85* gene from the *pMD18T-env* recombinant vector (expected size 930 bp) is shown in Figure 1A. The amplification of the *pgsA* gene from the *T7-pgsBCA* recombinant vector (expected size 1,143 bp) is shown in Figure 1B.

### Confirmation of the Recombinant pgsA-gp85 Protein in *L. plantarum*

**Figure 2:**
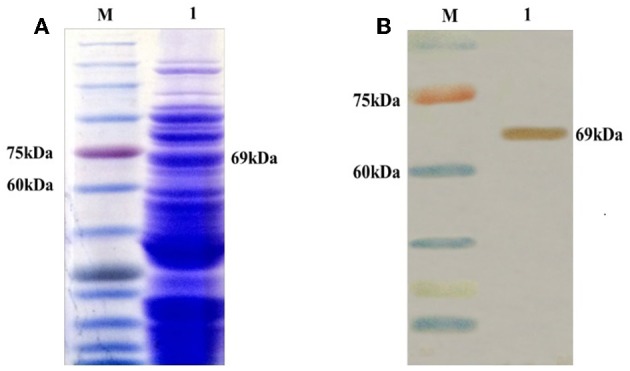
Identification of the recombinant pgsA-gp85 protein (unpurified) expressed in *L. plantarum* (pMG36e) by SDS-PAGE **(A)** and Western blot **(B)**. M: protein marker; 1: the pgsA-gp85 protein predicted as 69 kDa.

The recombinant gene *pgsA-gp85* was expressed in *L. plantarum*, and confirmed by SDS-PAGE (Figure 2A) and western blotting using the gp85-specific MAb (Figure 2B).

### Identification of Location of the Recombinant Fusion Protein

**Figure 3:**
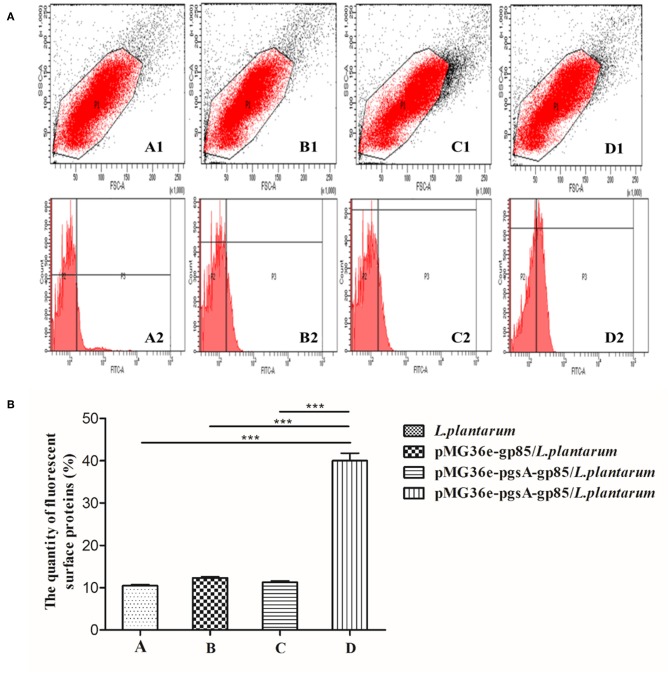
The results of flow cytometry analysis, monoclonal antibody JE9 as first antibody, FITC-conjugated anti-mouse IgG (Sigma) as secondary antibody to detect the A, B, and D samples and anti-mouse IgG (Sigma) as secondary antibody to detect the C sample. A: *L. plantarum*; B: pMG36e-gp85/*L. plantarum*; C, D: pMG36e-pgsA-gp85*/L. plantarum*. The map of P1 represents the selected flora, P3 stands for the fluorescence intensity of the protein on the surface of *L. plantarum* **(A)**. Histogram shows the quantity of fluorescence surface proteins in P3 part of flow cytometry analysis **(B)**. *P* < 0.01 indicates a significant difference between the control and experimental group at the same immunized time. ^***^P < 0.001.

Flow cytometry was used to analyze the cell surface display of *L. plantarum* (Figure 3A). The cell surface-displayed pgsA-gp85 was performed using mouse JE9 antibody as the primary antibody and FITC-conjugated goat anti-mouse IgG as the secondary antibody. *L. plantarum* and *L. plantarum* cells harboring the plasmid *pMG36e-gp85* were used as control for flow cytometry. The cells displaying pgsA-gp85 showed a significantly greater intensity of fluorescence signals than the control cells. This result is consistent with the data shown in Figure 3B.

### Body Weight (BW)

One week after vaccination, the BW of chickens in recombinant *L. plantarum* group were significantly higher than control group at 49, 63, 70, 77, and 84 d (*P* < 0.05). Meanwhile, BW of chickens in the natural *L. plantarum* group were also higher than control, but without significant differences to the recombinant group.

### Antibody Level

**Figure 4:**
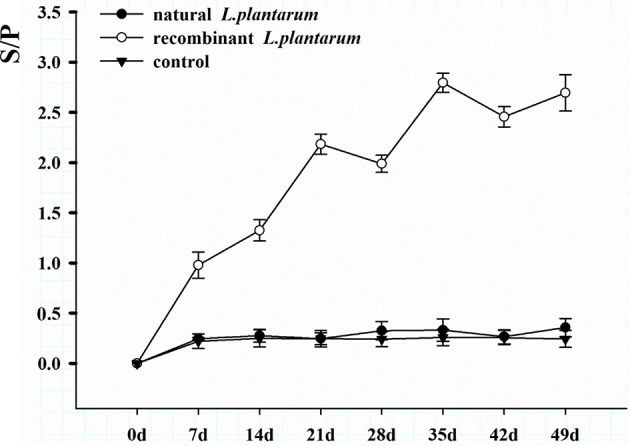
Changes of serum antibodies in different groups. The serum samples with a S/P value higher than 0.6 were considered ALV-J antibody positive. S/P: sample-to-positive = [(mean of OD_450nm_ of sample–mean of OD_450nm_ of negative control)]/[(mean of OD_450nm_ of positive control–mean of OD_450nm_ of negative control)].

**Figure 5:**
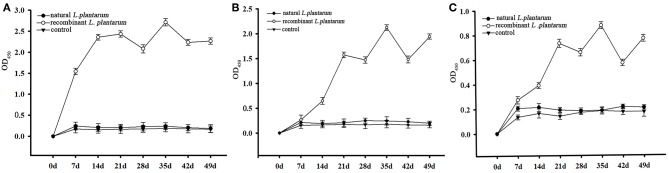
The levels of sIgA antibody. The *L. plantarum* group indicates the levels of IgA antibody in the chickens vaccinated with the *L. plantarum*, the recombinant *L. plantarum* indicates the levels of IgA antibody in the chickens vaccinated with the recombinant pMG36e-pgsA-gp85 *L. plantarum*, the control group indicates the levels of IgA antibody in the chickens vaccinated with the PBS. **(A)** The levels of sIgA antibody in bile samples. **(B)** The levels of sIgA antibody in duodenal lavages samples. **(C)** The levels of IgA antibody in serum samples.

Results showed that the recombinant pMG36e-pgsA-gp85 *L. plantarum* significantly triggered specific IgG and IgA antibodies against ALV-J, and enhanced the levels of IgG and sIgA compared to the control group (Figures 4, 5).

The level of systemic IgG in serum increased significantly after oral inoculation as shown in Figure 5. The sera of 15 chickens in the orally immunized recombinant *L. plantarum* group were taken at random intervals each weekly after Day 1 (pre-immune) and then analyzed by ELISA. A low level of IgG titers was detected after the primary immunization. The IgG level increased slowly after the first booster immunization, and increased statistically after the second booster immunization on Day 21 (3 weeks, *P* < 0.05). Peak IgG titers were reached after the third booster immunization on Day 35 (5 weeks). However, no significant specific antibodies were observed in the negative *L. plantarum* group until Day 49 the virus was challenged.

Samples were taken from three chickens in each group at random, at weekly intervals, from Day 1 (pre-immune) until Day 42 (6 weeks) and then analyzed by ELISA. The specific ALV-J IgA antibodies in bile, duodenal lavages and sera were detected during each test period (Table 1). The sIgA level increased consistently after oral inoculation in all samples of the recombinant *L. plantarum* group as shown in Figure 5.

**Table 1:** The positive ALV-IgA ratios in bile, duodenal lavages and serum samples of chickens from 7 to 49 d.

**Group**	**ALV-IgA positive ratio (%)**						
	**7 d**	**14 d**	**21 d**	**28 d**	**35 d**	**42 d**	**49 d**
Natural *L. plantarum*	0% (0/3)^a^	0% (0/3)^a^	0% (0/3)^a^	0% (0/3)^a^	0% (0/3)^a^	0% (0/3)^a^	0% (0/3)^a^
Recombinant *L. plantarum*	100% (3/3)^b^	100% (3/3)^b^	100% (3/3)^b^	100% (3/3)^b^	100% (3/3)^b^	100% (3/3)^b^	100% (3/3)^b^
PBS	0% (0/3)^a^	0% (0/3)^a^	0% (0/3)^a^	0% (0/3)^a^	0% (0/3)^a^	0% (0/3)^a^	0% (0/3)^a^

*Mean values at the same time point without a common superscript (a, b) differed significantly (P < 0.05)*.

Results of the sIgA levels in bile (Figure 5A) showed that, compared to the control groups (groups 1 and 3), the immunized group (group 2) started to increase rapidly after 7 days of the first immunization (*P* < 0.01). The profile showed a steady increase before Day 21 (3 weeks) and achieved the highest level on the 35th day after the 3rd booster immunization (5 weeks, *P* < 0.01). There were no significant differences between the *L. plantarum* group and PBS control group (*P* > 0.05).

The results of the sIgA level in duodenal lavages and sera (Figures 5B,C) showed that, compared to the control groups (group 1 and 3), there was a significant increase in the immunized group (group 2) from the 21st day after 2nd booster immunization. It also reached the highest level on the 35th day (5 weeks, *P* < 0.01). In addition, there was no significant difference between the *L. plantarum* group and PBS control group (*P* > 0.05). Therefore, these findings indicated that the recombinant pMG36e-pgsA-gp85 *L. plantarum* significantly enhanced the sIgA antibody response and produced local mucosal immune responses.

### Viremia

Viremia were determined weekly after challenge until the 77th day, the positive viremia ratios were calculated, and the results were shown in Table 2. The positive ratio of viremia in the natural *L. plantarum*+ALV-J and PBS+ALV-J groups were obviously higher than those in the recombinant *L. plantarum*+ALV-J group.

**Table 2:** ALV-J positive viremia ratios in the chickens from 1 to 4 weeks after being challenged with ALV-J.

**Group**	**ALV-J positive ratio (%)**			
	**1st week**	**2nd week**	**3rd week**	**4th week**
Natural *L. plantarum*+ALV-J	16.7% (2/12)^a^	25.0% (3/12)^ac^	41.7% (5/12)^a^	41.7% (5/12)^a^
Recombinant *L. plantarum*+ALV-J	8.3% (1/12)^a^	8.3% (1/12)^ab^	25.0% (3/12)^ab^	16.7% (2/12)^ab^
PBS+ALV-J	33.3% (4/12)^a^	50.0% (6/12)^c^	80.0% (8/10)^c^	80.0% (8/10)^c^

*Mean values at the same time point without a common superscript (a–c) differed significantly (P < 0.05)*.

## Discussion

Before 2005, ALV-J was widely recognized and reported in many regions of the world; it was predominantly prevalent in white feather broiler chickens (1, 25, 26). However, in recent years, ALV-J has also occurred in commercial layer flocks (27), and it has become a serious threat to local breeder flocks. For many pathogens, initial infection occurs in the mucosa of animals. Chickens with ALV-J can infect other healthy individuals via their excrements or cloacal secretions. Therefore, our study was devoted to seeking an effective vaccine that would protect chickens from ALV-J infection. Compared to routine styles of antigen delivery, mucosal immunization still offers some advantages including those of being more convenient, lower cost, and less stress (14, 15). In addition, lactic acid bacteria have been proposed as a live vehicle for the delivery of exogenous antigen proteins for mucosal immunization or for other therapeutic molecules. The biological functions of lactic acid bacteria are effectively combined with the immunogenicity of exogenous antigen genes (19, 20, 28).

*L. plantarum*, a lactic acid bacteria, that can be used as a probiotic for animal gastrointestinal tracts, has many functions, such as maintenance of intestinal flora, enhancement of immunity, and promotion of nutrient absorption. *L. plantarum* can also act as a potential delivery vehicle for mucosal vaccines because it is generally regarded as safe and is able to protect antigens from premature disintegration and degradation in animal gastrointestinal tract (19, 20).

However, the inherent immunogenicity of vaccine antigens in many cases is insufficient to elicit an effective immune response, which led to the suggestion that displaying antigens on the bacterial surface maybe a practical method for enhancing antigen immunogenicity (29). To this end, ALV-J gp85 protein was expressed on the surface of *L. plantarum* using the surface-display motif, *pgsA*, which was adopted from the poly-γ-glutamic synthetase complex. By using this approach, we hoped to address the aforementioned challenges. In the present study, the results indicated that *pgsA* effectively displayed the gp85 protein on the surface of *L. plantarum* (Figures 3, 4). *L. plantarum* as a carrier can successfully express exogenous antigens, resist damage from the extreme gastrointestinal environment, and significantly enhance the immunogenicity of pgsA-gp85 antigens. Thus, it plays a double role of adjuvant and vector of the recombinant vaccine.

Because ALV-J is able to cause growth retardation in infected chickens, body weight is considered as a valuable indicator to evaluate the protective effect of our established recombinant vaccine (30, 31). As expected, in our study, body weight in the recombinant *L. plantarum* group was significantly higher than that in the control group (*P* < 0.05) at 49, 63, 70, 77, 84 d, indicating that vaccination with recombinant *L. plantarum* can prevent body weight loss caused by ALV-J infection in chickens.

This study described the mucosal and systemic immune responses specific to ALV-J induced by an orally administered recombinant *L. plantarum* vaccine. Efficient mucosal immunity is mediated predominately by secretory IgA (32, 33). Some studies also show that recombinant *L. plantarum* can induce an intestinal mucosal immune response, promote the secretion of intestinal mucosal sIgA, and strengthen intestinal mucosal immunobarrier function (34).

IgA is the predominant antibody on the mucosal surface, as it is produced locally at a level that exceeds those of all other immunoglobulins (35, 36). Therefore, an efficient gp85 oral vaccine can induce a specific mucosal immune response of IgA (Table 1). In our study, we found that the sIgA levels in bile, duodenal lavages, and sera of the vaccinated chickens were dramatically increased after the first immunization compared to the natural *L. plantarum* and PBS groups, it reached the highest level on the 35th day after the third booster immunization (*P* < 0.01). These results indicated that recombinant *pMG36e-pgsA-gp85 L. plantarum* effectively enhanced the sIgA antibody response and produced strong local mucosal immune responses (Figure 5). Thus, *L. plantarum* could act as an excellent mucosal adjuvant and vector of recombinant vaccines (37).

Moreover, mucosal delivery of vaccines should also elicit specific immunity in systemic lymphoid tissues because most infections that gain entrance through mucosal surfaces will become systemic (38–40), and in this study recombinant *L. plantarum* can induce high IgG in sera. IgG is the main antibody produced by the humoral immune response, and it plays an important role against infection, including neutralizing toxins and internal conditioning in body defense mechanisms (41). Some researchers have succeeded in detecting the specificity of IgG by using recombinant lactic acid bacteria to express exogenous genes (42, 43).

IgG is an important indicator for assessing the immunity efficiency of exogenous vaccines. In our study, results showed that IgG titers of the recombinant *L. plantarum* group were significantly higher than those in the natural *L. plantarum* and control group from Day 7 to 49 (Figure 4). In addition, further study of ALV viremia was performed to verify the protection created by this vaccine. Compared to the natural *L. plantarum* and control groups, after challenge, better protection was observed in the recombinant *L. plantarum* group against ALV-J.

In conclusion, our study demonstrated that the ALV-J gp85 protein was present on the surface of the non-pathogenic *L. plantarum*, this can be administered orally to animals, tolerate gastric acidity, elicit both mucosal and systemic immune responses, and effectively alleviate viremia.

## Author Contributions

JL and YL designed the study. SW, YX, and NG contributed analytic tools. SW, YQ, and NG analyzed the data. JL, KL, and MS wrote the paper. DZ, YL, and MS revised the manuscript. All authors reviewed the results and approved the final version of the manuscript.

### Conflict of Interest

The authors declare that the research was conducted in the absence of any commercial or financial relationships that could be construed as a potential conflict of interest.
